# EFFECTS OF FLAVANOL (+)-EPICATECHIN ON SKELETAL MUSCLE OF AGED RATS

**DOI:** 10.1093/geroni/igac059.2890

**Published:** 2022-12-20

**Authors:** Israel Ramirez-Sanchez, Viridiana Navarrete, Leonor Galera, Francisco Villarreal

**Affiliations:** University of California San Diego, Mexico City, Distrito Federal, Mexico; IPN, Mexico City, Distrito Federal, Mexico; IPN, Mexico City, Distrito Federal, Mexico; UCSD, San Diego, California, United States

## Abstract

Sarcopenia is a progressive and generalized age-related skeletal muscle (SkM) disorder characterized by the accelerated loss of muscle mass (atrophy) and function. SkM atrophy is associated with increased incidence of falls, functional decline, frailty and mortality. In its early stage, SkM atrophy is associated with oxidative stress, increased pro-inflammatory cytokine levels and proteasome-mediated protein degradation. These processes also link to the activation of atrophy associated factors and signaling pathways which lack any approved, targeted pharmacotherapy. We have characterized the capacity of flavanols specifically, epicatechin to favorably modulate SkM mass and function in animal models of SkM disease and in proof of concept studies in humans including muscular dystrophies. In this study we wished to evaluate the capacity of this flavanol to ameliorate aging associated sarcopenia. Using 24-month-old male rats a 4 week oral administration of the flavanol (+)-epicatechin (1mg/kg/day) was implemented while control rats only received vehicle (water). SkM strength (grip) and endurance (treadmill walk), muscle size, (muscle mass, cross sectional area of fibers), ubiquitin-proteasome atrophic pathway (including myostatin, Murf1 and Atrogin1 by Western blots) and markers of oxidative stress and inflammation in gastrocnemius were quantified. We also analyzed a relevant protein synthesis pathway (IGF/AKT/mTOR). The use of (+)-epicatechin in aged rats led to significant changes as follows: 1) improved SkM mass and function, 2) downregulation of atrophic pathway, 3) reduced levels of oxidative stress and proinflammatory cytokines and, 4) upregulation of protein synthesis pathway. Given its safety and tolerance profile this agent warrant its rigorous investigation in clinical trials.

